# Concomitant Use of Herbal and Conventional Medicines among Patients with Diabetes Mellitus in Public Hospitals of Addis Ababa, Ethiopia: A Cross-Sectional Study

**DOI:** 10.1155/2020/4871459

**Published:** 2020-06-15

**Authors:** Solomon Getnet Meshesha, Mariamawit Yonathan Yeshak, Gebremedhin Beedemariam Gebretekle, Zelalem Tilahun, Teferi Gedif Fenta

**Affiliations:** ^1^Addis Ababa Regional Health Bureau, Addis Ababa, Ethiopia; ^2^Department of Pharmacognosy and Pharmaceutical Chemistry, School of Pharmacy, College of Health Sciences, Addis Ababa University, Addis Ababa, Ethiopia; ^3^Social and Administrative Pharmacy Working Group, Department of Pharmaceutics and Social Pharmacy, School of Pharmacy, College of Health Sciences, Addis Ababa University, Addis Ababa, Ethiopia

## Abstract

**Introduction:**

The majority of the population in developing countries including Ethiopia still relies on traditional medicines (TMs). Patients with chronic illness like diabetes mellitus (DM) are dissatisfied with conventional medicines and thus are more likely to simultaneously use herbal medicines (HMs). However, such practice could result in potential herb-drug interaction. This study aimed to identify the commonly used HMs among patients with DM and determine the magnitude of concomitant use of herbal and conventional antidiabetic medicines.

**Method:**

A health facility-based cross-sectional study design was employed using both quantitative and qualitative data collection methods to determine the magnitude of concomitant use. Patients with DM and prescribers from four public hospitals were the study population for the quantitative and qualitative study, respectively. Simple descriptive statistics were used to describe variables for the quantitative data, and content analysis had been conducted manually for qualitative data.

**Result:**

Out of 791 respondents, 409 (51.7%) used traditional medicine at least once in their life time, and 357 (45.1%) used traditional medicine in the last six months prior to data collection. A majority (288 (80.7%)) of the respondents used HMs after starting the conventional antidiabetic medicines within the last six months. *Moringa stenopetala*, *Thymus vulgaris*, *Trigonella foenum-graecum*, *Nigella sativa*, and *Allium sativum* were among the frequently mentioned HMs. Prescribers were requesting patients' HM use when they saw sign of liver toxicity and skin disease, and they were not documenting their history in the patient's chart.

**Conclusion:**

Concomitant use of herbal and conventional antidiabetic medicines was a common practice. Cognizant of its potentially serious herb-drug interactions, efforts should be made to improve awareness and knowledge of healthcare providers about HM potential effects. Further studies on dose, frequency, duration, and modes of interaction are recommended.

## 1. Introduction

Traditional medicines (TMs) cover a heterogeneous spectrum of ancient to new-age approaches to prevent or treat disease. They include the use of herbal medicines (HMs), spiritual healing, and practices such as bone setting [1]. TMs were used for age-related chronic diseases such as hypertension and diabetes mellitus (DM) for which no modern medicine or only palliative therapy is available [2]. The use of HMs, either alone or combined with conventional medicines, has been practiced widely particularly in low-resource setting countries. Their use in patients with chronic disease like DM is very common, owing to its natural origin and perceived lesser side effects or dissatisfaction with conventional medicines [3–5].

DM is one of the largest global health emergencies of the 21st century. In 2015, 14.2 million people were estimated to be living with DM in Africa, and this is projected to 34.2 million by 2040 [6]. The prevalence of DM in Ethiopia among adults aged 20–79 was estimated to be 0.2% and 0.28% in 2010 and 2030, respectively, with mean annual increment 0.06% [7]. Literature showed that patients with DM who were dissatisfied with conventional medicine were more likely to use HM to enhance the effect of conventional treatment [8]. However, simultaneous use of herbal and conventional medicines may result in potentially serious herb-drug interactions leading to negative effect on patient outcomes [4]. Mostly, HMs are complex mixtures of more than one active ingredient which increase the possibilities of interactions [9, 10]. For instance, treatment with St. John's wort (SJW) significantly increases the apparent clearance of gliclazide [11]; administration of *Ginkgo biloba* extract increased hepatic clearance of insulin and oral hypoglycemic agents, hence resulting in an increased glucose level [12]. Adverse effects such as hypoglycemia and lactic acidosis were also detected among patients with diabetes who were taking herbal antidiabetic products in China [13]. Such adverse reactions could involve all systems, age groups, and severity [9].

Until recently, however, herb-drug interaction was often unsuspected by healthcare providers. Most trained physicians lack adequate knowledge on HM and its potential for drug interactions [14]. Patients may also not always inform their doctor of the simultaneous use of HM [15].

Ethiopia is not an exception to this worldwide phenomenon as HM is still the main source of healthcare for many millions of people [16]. However, information on most commonly used HM and extent of concomitant use with conventional medicines in DM is scanty. This study aimed to determine the extent of concomitant use of herbal and conventional antidiabetic medicines and also to identify the commonly used HMs among patients with DM.

## 2. Methods

### 2.1. Description of the Study Area

The study was conducted in Addis Ababa, the capital city of Ethiopia. Addis Ababa is divided administratively into 10 subcities and has a total population of 3.43 million [17]. 56 hospitals (11 public, 3 army, and 42 private hospitals), 760 private clinics, 96 health centers, and 720 pharmacies were available in the city administration [18].

### 2.2. Study Design

A health facility-based prospective cross-sectional study design was carried out to determine the prevalence of concomitant use of herbal and conventional medicines among patients with DM. Data were collected between April and August, 2016.

### 2.3. Source and Study Population

The source population for the quantitative data constituted all patients with diabetes attending treatment in outpatient clinics of public hospitals in Addis Ababa City Administration (AACA). Patients with diabetes from four public hospitals in Addis Ababa, two hospitals from AACA (Yekatit 12 Hospital Medical College and Zewditu hospital) and two hospitals from Federal (St. Paul's Hospital Millennium Medical College (SPHMMC) and Tikur Anbessa Specialized Hospital) were the study population. On the contrary, all prescribers working in the diabetic clinics were considered as the source population for the qualitative data.

### 2.4. Inclusion and Exclusion Criteria

Patients aged ≥18 years and taking antidiabetic medicines were eligible for the survey. Patients with DM who were physically or mentally not capable for the interview and those who were health professionals were excluded from the study. For the qualitative interview, prescribers who were working in diabetic clinics and had no previous diagnosis of DM were recruited.

### 2.5. Sampling and Sample Size Determination

The sample size was determined using the single population proportion formula [19]. A total of 845 patients with DM were included by considering 50% proportion of concomitant use of herbal and conventional medicine, with 95% confidence interval, 5% margin of error, design effect of 2, and 10% nonresponse rate.

Based on ownership, hospitals were stratified into those administered by AACA and Federal Government. Then, from each stratum, two hospitals, Zewditu Memorial Hospital (ZMH) and Yekatit 12 Hospital Medical College, from AACA and Tikur Anbessa Specialized Hospital (TASH) and St. Paul's Hospital Millennium Medical College (SPHMMC) from federal hospitals were selected using simple random sampling technique. The sample size was proportionately allocated considering total patients with diabetes of each hospital of which 336 were from TASH, 206 from SPHMC, 132 from ZMH, and 171 from Yekatit 12 Hospital Medical College. Recruitment of the respondents was achieved through the consecutive sampling approach.

For the qualitative data, selection of participants was based on the purposive sampling method. Key informants were recruited on the basis of their professional role and work experience in the diagnosis and treatment of diabetes mellitus. Selection of participants was continued until theoretical saturation was achieved.

### 2.6. Data Collection Instrument

For the concomitant use of herbal and conventional medicine assessment, a total of 45-item questionnaire was adopted from the previous literature (supplementary 1) [20]. Questions addressing the patients' sociodemographic characteristics (age, sex, average income, marital status, and educational status), traditional medicine use, type of traditional medicine chosen, time of use of traditional medicine (before, after, or during the conventional medicine treatment), reason to use the traditional medicine, perceived effectiveness of the herbal medicine, and adverse effects experienced were included in the assessment questionnaire.

On the contrary, a semistructured open-ended interview guide with flexible probing techniques was employed to explore prescriber's experience of history taking about herbal medicine use during examination of patients with DM.

### 2.7. Data Collection Procedures

Two trained pharmacists were recruited as data collectors for each hospital. As part of the training, data collectors conducted pretesting of the instrument in Ras Desta Damtew Memorial Hospital under the supervision of the principal investigator, and modifications were done accordingly. All key informant interviews were administered by the principal investigator, who was trained on qualitative research methods. All interviews were recorded, and notes were also taken during interviews to expand later. The interviews were done in Amharic (a local language), and any ambiguities raised from the interviewees were cleared at the time of the interview. Questionnaires prepared in English were translated into Amharic and backtranslated into English to ensure consistency.

### 2.8. Data Analysis

After the data collection, the principal investigator coded each question, and data entry was made using Epi Info version 3.5.4 and transferred to SPSS version 20 for analysis. Descriptive statistics were used to summarize the data.

The qualitative data were analyzed using content analysis. It involved intensive reading and rereading through the data, and content analysis had been conducted manually, focusing on similarities and differences of perspectives between different informants.

### 2.9. Ethical Consideration

Ethical approval was obtained from the Ethics Review Committee of the School of Pharmacy, Addis Ababa University, and AACAHB Ethics Review Board. The study was conducted after obtaining permission from the respective hospitals. Participants of the study were also asked for verbal consent before participating in the study. Participants were assured about confidentiality of the information obtained in the course of the study, and there was no use of personal identifiers.

## 3. Results

### 3.1. Quantitative Findings

#### 3.1.1. Demographic Characteristics of Respondents

From 845 respondents, 791 completed the interview, making up a response rate of 93.6%. Males and females accounted almost similar proportion of sample size of 402 (50.8%) and 389 (49.2%), respectively. The age of the respondents ranged from 18 to 83 with the mean age of 49.07 ± 14.76 years. Among the respondents, one-third of them were housewives, and two-thirds of respondents had attended at least elementary school. A majority (667 (84.3%)) of the respondents were reported as residents of Addis Ababa (Table 1).

#### 3.1.2. Clinical Condition of Respondents

At the time of the study, 210 (26.5%) of patients were on insulin injection, 499 (63.1%) on oral hypoglycemic medicines, and 82 (10.4%) on both. A sizeable number of the patients (333 (42.1%)) were diagnosed as diabetic before 5 to 10 years. More than half (58%) of them had other comorbid conditions, of which 313 (39.5%) of patients had hypertension (Table 2).

#### 3.1.3. Traditional Medicine Use Practice of Patients with DM

More than half (409 (51.7%)) of the respondents revealed their use of TM at least once in their life time, and 357 (45.1%) used TM in the last six months. Of these, the majority (218 (61.1%)) of them was used to treat only diabetes mellitus. From the total TM users within the last six months, 288 (80.7%) used HMs.

Patients with diabetes mentioned various reasons for using TM. The most common one was patient's belief that TM is more potent than conventional antidiabetic medicines which was mentioned by 240 (58.7%) of the respondents. A sizable number of respondents (96 (23.5%)) also believed conventional medicine is expensive and reported to use TM (Table 3).

#### 3.1.4. Source, Duration, and Part of the Herb Used

The majority (260 (90.3%)) of patients remembers the name of the herbal medicine they used, and 211 (73.3) of them reported using the leaf of the herb. Market 217 (75.3%), garden 49 (17%), and traditional healers 31 (10.8%) were the major sources for obtaining herbal medicine. From the total herbal medicines, 111 (38.5%) respondents used herbal medicine once, and 40 (13.9%) respondents used for more than 30 days (Table 4).

#### 3.1.5. Commonly Used Herbal Medicines

*Moringa stenopetala* was used by 144 (50%) of the patients with DM. Similarly, *Thymus vulgaris*, *Trigonella foenum-graecum*, *Nigella sativa*, and *Allium sativum* were reported by 50 (17.4%), 21 (7.3%), 19 (6.6%), and 16 (5.6%) of patients with DM, respectively. A considerable amount of herbal medicines was prepared in crushed form as cited by 194 (67.4%) of patients, and 243 (84.4%) of them expressed that the herb was available in dried form. Oral administration was the major route of herbal medicines used among patients with DM (Table 5).

#### 3.1.6. Source of Information and Decision on Herbal Medicines Used

Of 288 herbal medicine users, 263 (91.3%) respondents tried to get more information about the HM related to its effectiveness and toxicity they used (Table 6). Of those, 164 (62.4%) of them obtained the information from the community, and only 3 (1.2%) respondents obtained it from pharmacists. Of the total 288 concomitant users of herbal and antidiabetic conventional medicines, only 103 (35.8%) of them informed their healthcare providers that they were using HMs. Being afraid of the doctor and prevailed perception that HM has no problem were the frequently mentioned reasons for failing to inform their healthcare providers (Table 6). Among those who informed their use of HM to health professionals, 74 (71.8%) of them were counseled to continue the herbal medicine, while 28 (27.2%) were advised to discontinue (Table 6).

#### 3.1.7. Perception of Effectiveness and the Method of Side Effect Aversion

From 288 respondents, 40 (13.9%) of them had encountered side effects from HM use. Among these, constipation, vomiting, and headache were the most frequently reported side effects as mentioned by 14 (24.6%), 8 (20%), and 8 (20%) of the participants. Of those who had side effects, 24 (60%) discontinued herbal medicine use by themselves, and 10 (25%) consulted health professionals to avert the side effects (Table 7).

### 3.2. Qualitative Findings

A total of 8 physicians (4 general practitioners and 4 consultants) were interviewed. Five of them were males, and their mean age was 44.2 (ranged from 29 to 54 years). Participants' work experience in diabetic clinics was from 2 to 12 years.

Three major themes were defined in qualitative data analysis: prescriber's perceived knowledge about herb-drug interactions, herbal medicine history taking practice, and commonly used herbal medicines.

#### 3.2.1. Prescribers' Perceived Knowledge about Drug-Herb Interaction

The majority (*n* = 6) of key informants agreed on the importance of knowing most commonly used herbal medicines and their effect when used concomitantly with conventional medicine. Nevertheless, all (*n* = 8) of them revealed that they had no adequate knowledge about herb-drug interactions. They also reported that much emphasis was not given to the herb-drug interaction course in medical schools. As a result, low attention was given to taking history of herbal medicine use. One general practitioner strengthened this as

“If I ask the patient whether he/she has been using herbal medicine and found that he/she was using any herb, I wouldn't take any decision towards using herb because I don't have any knowledge about a specific herb and the conventional anti-diabetic drug interactions.”

#### 3.2.2. Herbal Medicine History Taking Practice

All (*n* = 8) key informants mentioned that documentation of herbal medicine information for patients taking conventional medicines was not usual practice. On the contrary, a majority (*n* = 5) of them claimed of asking patients about their herbal medicine experience when they saw perceived side effects like sign of liver toxicity. Lack of knowledge about effects of concomitantly using herbal and conventional medicines and high patient load were ascribed as major reasons for this. This was strengthened by one of the participants as follows:

“Usually, I don't check patients' history of herbal medicine use practice. I haven't learnt it at school and I don't have experience on how to handle it. But even if I want to do it, there are a lot of patients waiting for service so that I don't have time to ask my clients about their herbal medicine use practice but occasionally when I suspect signs of liver toxicity, I would ask whether they took herbal medicine or not. But, I would not record it on the patients' chart.”

#### 3.2.3. Commonly Used Herbal Medicines

Most of the key informants (*n* = 7) agreed that *Moringa stenopetala* is the most commonly used herb by patients with diabetes as it was believed to have hypoglycemic effect. Respondents perceived that concomitant use of herbal-conventional medicines was a prevalent practice, but they found it difficult to remember the other types of herbs used mentioned by the patient. Only one physician had encountered patients who were using *Coffea arabica* extract for diabetic treatment.

One general practitioner also explained that there were patients who told him as they were using herbal medicines for the treatment of diabetes mellitus. He expressed his experience as follows:

“There were plants used by patients with diabetes that I didn't remember the name and type of the herbs since I did nothing with that. I only advised them to discontinue using herbal medicine.”

## 4. Discussion

This study has been carried out with the purpose of determining the prevalence of herbal medicine use and identifying the commonly used herbs by patients with DM. The study estimated that, from total patients with DM, 288 (36.4%) took herbal medicine which was higher than a study in black South African communities, where 28 (21%) used HM to treat hypertension [21]. This difference could be due to the clinical difference of patients included in the study. On the contrary, our finding was lower than the prevalence in Gucha district, Kenya (68.9%), and southeast region of Morocco (80%) [22, 23]. The difference in magnitude may be due to difference in the study population.

In this survey, 231 (69.6%) of the respondents used HMs along with prescribed therapy for the last six months which was higher than in South Sudan (58%) [24]. The higher concomitant use in this study might be due to the difference in sociodemographic characteristics of the study populations between the two studies. Similarly, a higher prevalence was also obtained from India, where 71% of patients with DM used HMs along with the conventional therapy [8]. The higher use might be due to better integration of HMs with the modern healthcare system in India [25].

Even though the quantitative study indicated high proportion of patients with diabetes used HMs concomitantly with conventional antidiabetic medicines, finding on exploration of HM history taking practice of prescribers during physical examination of patients with diabetes showed low. The contradictory results from the quantitative and qualitative data could be due to prescriber's low knowledge on herb-drug interaction and work load.

*Moringa stenopetala*, *Thymus vulgaris*, *Trigonella foenum-graecum*, *Nigella sativa*, and *Allium sativum* were the most frequently used HMs as claimed by the participants in this study. A similar study in Oriental Morocco also indicated that the most five HMs used by Morocco population were *Salvia officinalis*, *Trigonella foenum-graecum*, *Olea europaea*, *Artemisia herba*-*alba*, and *Origanum vulgare* [26]. Another similar study in Turkey revealed that the most frequently used HMs by the patients are green tea, garlic, and ginger [27]. Different HMs might be used in different areas with different levels as a result of difference in accessibility of the herb and information dissemination among patients with diabetes. *Moringa stenopetala* and *Coffea arabica* extract were also identified as the commonly used HMs by diabetic patients in the qualitative study.

A majority of patients with DM (240 (58.7%)) in this study used HM since they believed that TM was more potent and affordable than conventional antidiabetic medicine which was the major reason to use HM. Similarly, studies in North Sudan [24] and Kenya [28] indicated patients' belief in herbal medicine efficacy, easy accessibility, and lower cost have encouraged patients with diabetes to use them.

This study also revealed that 69.1% of the HM users perceived herbal medicines they used were effective. Qualitative data also indicated patients with diabetes were benefited from *Moringa stenopetala*. Scientific experimental studies on *Moringa stenopetala* leaves [29], *Allium sativum* [30], *Thyme vulgaris* [31], *Trigonella foenum-graecum* [32], and *Nigella sativa* [33] had showed significant decrease in blood glucose level. Another study also revealed that fasting blood glucose was reduced by *Zingiber officinale* to normal as effectively as glibenclamide [34] and *Allium sativum* as compared to metformin [35].

This study also revealed that 40 (13.9%) of HM users reported they experienced side effects. This might be due to an additive and/or synergetic effect of the concomitant use of these herbs with conventional antidiabetic medicines. Like hypoglycemic effect, experimental studies on *Trigonella foenum-graecum* [36, 37], *Nigella sativa* [38], and *Moringa stenopetala* [39] identified different toxic effects.

This study revealed that the very common method of remedy preparation was in dried and crushed form which was taken by mixing with water. However, another study in Ethiopia indicated more than half of the HMs were used in fresh form [40], and decoction was the most common method of preparations [41]. Even though oral administration was the major route and leaf of the herb was the most frequently used 211 (73%) parts of the herb in this study, different route of administration and various parts of the herb in different preparation were also used. Using different route of administration, preparation and parts of the herb may yield different active ingredients of a herb which might result in different effect [42].

This study found that market and community were the major sources and sources of information for the HMs, respectively. The societies' belief on antidiabetic effect of HMs might affect adherence to the conventional medicine. This survey also indicated that a small proportion of respondents obtained information about the HMs from healthcare providers. This result was supported from Ubon Ratchathani province, Thailand [43]. Lack of free communication between prescribers and patients with diabetes might be the reason.

## 5. Conclusion

According to the findings of this study, herbal medicines are used concomitantly with conventional antidiabetic medicines for the treatment and management of DM by patients attending treatment in four selected hospitals of Addis Ababa. More than eighty percent of patients with diabetes used HMs, and 231 (69.6%) of them used HMs concomitantly with conventional antidiabetic medicines. Combined use of HMs and conventional antidiabetic medicines could potentially lead to serious toxic effects due to herb-drug interactions. *Moringa stenopetala*, *Thymus vulgaris*, *Trigonella foenum-graecum*, *Nigella sativa*, *Allium sativum*, *Linum usitatissimum*, *Zingiber officinale*, *Ocimum lamiifolium*, *Brassica nigra*, *Ruta chalepensis*, and *Camellia sinensis* were the most commonly used herbs by patients with diabetes.

## Figures and Tables

**Table 1:** Demographic characteristics of respondents in selected public hospitals of Addis Ababa, Ethiopia, 2016 (*n* = 791).

Variable	Frequency	Percent
Gender		
Male	402	50.8
Female	389	49.2

Age		
18–40	290	36.7
41–64	368	46.5
>64	133	16.8

Marital status		
Single	159	20.1
Married	526	66.5
Divorced	43	5.4
Widowed	63	8.0

Residency		
Addis Ababa	667	84.3
Oromia	111	14.1
Others^*∗*^	13	1.6

Religion		
Orthodox Christian	590	74.6
Muslim	97	12.3
Protestant Christian	89	11.2
Others^*∗∗*^	15	1.9

Educational level		
Unable to read and write	187	23.7
Nonformal education	80	10.1
Elementary school	166	21.0
High school	190	24.0
TVET college and above	168	21.2

Occupation		
House wife	231	29.2
Government employee	146	18.5
Merchant	70	8.8
Daily laborer	80	10.1
Student	45	5.7
Jobless	75	9.5
Others^*∗∗∗*^	144	18.2

^*∗*^Tigray, SNNP, and Amhara regions; ^*∗∗*^Jehova's Witness and Pagan; ^*∗∗∗*^retired, contract employee, and handicraft.

**Table 2:** Clinical conditions of patients with diabetes mellitus in selected public hospitals of Addis Ababa, Ethiopia, 2016 (*n* = 791).

Variable	Frequency	Percent
Current treatment		
Insulin	210	26.5
Oral hypoglycemic	499	63.1
Both	82	10.4

Comorbidity type^*∗*^		
Hypertension	313	39.6
Asthma	49	6.2
Kidney disease	65	8.2
Heart problem	53	6.7
Eye disease	47	5.9
Others^*∗∗*^	126	15.9
None	332	42.0

Years of pre-existing diabetes diseases		
<5	174	22.0
5–10	333	42.1
>10	284	35.9

^*∗*^Percentages will not add up to 100% as some DM patients had more than one type of comorbidity; ^*∗∗*^atherosclerosis, gastrointestinal problem, hemorrhoid, HIV/AIDS, neurological problem, mental problem, and so on.

**Table 3:** Traditional medicine use practices of patients with diabetes in selected public hospitals of Addis Ababa, Ethiopia, 2016 (*n* = 791).

Variable	Frequency	Percent
Ever use of TM		
Yes	409	51.7
No	382	48.3

TM use in the last six months		
Yes	357	87.3
No	52	12.7

Disease to be treated using TM		
Diabetes mellitus	218	61.1
Diseases other than DM	25	7.0
Both	114	31.9

Time for TM use		
Before starting antidiabetic drugs	101	30.4
After starting antidiabetic drugs	231	69.6

Type of TM		
Herb/herb product	288	80.7
Holy water	69	19.3

Perceived reasons to use TM		
TM is more potent and curable	240	58.7
High cost of the conventional drugs	96	23.5
Toxicity/side effect of the conventional drugs	33	8.1
Inaccessibility to the modern health facilities	29	7.1
Others^*∗*^	11	2.7

^*∗*^Others' reasons to use TM include unsatisfactory service in health facilities, to augment treatment, and no reason.

**Table 4:** Source, duration, and part of the herb used by respondents in selected public hospitals of Addis Ababa, Ethiopia, 2016 (*n* = 288).

Variable	Frequency	Percent
Remember the name		
Yes	260	90.3
No	28	9.7

Part of the herb used^*∗*^		
Leaf	211	73.3
Seed	37	12.8
Oil	19	6.3
Root	5	1.7
Stem	4	1.4
Others^*∗∗*^	65	22.6

Source of the herb^*∗*^		
Traditional healer	31	10.8
Market	217	75.3
Garden	49	17
Others^*∗∗∗*^	24	8.3

Duration of use		
Used once	111	38.5
2–7 days	57	19.8
8–30 days	80	27.8
More than 30 days	40	13.9

^*∗*^Multiple responses were possible. ^*∗∗*^Do not know the part, fruit, and flower. ^*∗∗∗*^Neighbor, family, and another user.

**Table 5:** Commonly used herbal medicines by patients with diabetes in selected public hospitals of Addis Ababa, Ethiopia, 2016 (*n* = 288).

Local name	Common name	Scientific name	Part used	Frequency^*∗*^	Percent	Formulation
Shiferaw	Moringa	*Moringa stenopetala*	Leaf	144	50	Dried leaves of Moringa are crushed and taken as tea on daily basis.
Tosign	Thyme	*Thymus vulgaris*	Leaf	50	17.4	Tea of dried, crushed leaves is taken daily.
Abish	Fenugreek	*Trigonella foenum-graecum*	Seed	21	7.3	Dried seeds are powdered. The powder is mixed with water and taken in the morning with an empty stomach.
Tikurazimud	Black seed	*Nigella sativa*	Oil	19	6.6	Oil obtained from squeezed seeds of the dried black seed is taken after food at any time.
Nech Shinkurit	Garlic	*Allium sativum*	Knob/clove	16	5.6	Fresh knob or clove of garlic cooked with food.
Teliba	Linseed	*Linum usitatissimum*	Seed	9	3.1	Dried seeds of Linseed are powdered. The powder of linseed seed is mixed with water and taken with food.
Zingible	Ginger	*Zingiber officinale*	Rhizome	9	3.1	The dried and crushed rhizomes of ginger are cooked with food.
Damakese	Damakese	*Ocimum lamiifolium*	Leaf	9	3.1	Fumigation of the fresh leaf.
Senafich	Mustard	*Brassica nigra*	Seed	7	2.4	Dried seeds are powdered. The powder is then mixed with water and taken with food.
Tena'dam	Rue	*Ruta chalepensis*	Leaf	5	1.7	Fresh leaves are soaked in tea for some time and drunk.
Arenguade Shay kitel	Green tea	*Camellia sinensis*	Leaf	3	1	The dried leaves of green tea are soaked in hot water for some time and drunk as tea.
Others^*∗∗*^				28	9.7	

^*∗*^Multiple responses were possible. ^*∗∗*^Do not know the name and part of the herb.

**Table 6:** Source of information for decision on herbal medicine use of diabetic patients interviewed in selected public hospitals of Addis Ababa, Ethiopia, 2016 (*n* = 288).

Variable	Frequency	Percent
Sought for more information		
Yes	263	91.3
No	25	8.7

Recommendation obtained from		
Community	164	62.4
Prescriber	24	9.1
Herbalist	28	10.6
Pharmacist	3	1.1
Others	44	16.7

Patients who informed the doctor		
Yes	103	35.8
No	185	64.2

Reason not to inform the healthcare provider		
Afraid of the doctor	74	25.8
There is no problem	72	25.0
The prescriber did not ask	14	4.9
Considered as food	25	8.7

Has information about drug-herb interaction		
Yes	44	15.3
No	244	84.7

Prescribers' advice		
Discontinue	28	27.2
Continue	74	71.8
Neither continue nor discontinue	1	1.0

**Table 7:** Herbal medicines' effectiveness perception and the method of side effect aversion of respondents in selected public hospitals of Addis Ababa, Ethiopia, 2016 (*n* = 288).

Variable	Frequency	Percent
Effectiveness perception		
Yes	199	69.1
No	89	30.9

Side-effect experience		
Yes	40	13.9
No	248	86.1

Type of side effects^*∗*^		
GI problem	5	12.5
Vomiting	8	20.0
Headache	8	20.0
Diarrhea	5	12.5
Tiredness	5	12.5
Constipation	14	35.0
Abdominal pain	5	12.5
Others	8	20.0

Actions to avert side effect		
Consulted the health professional	10	25.0
Discontinued using herbal medicine	24	60.0
Consulted the herbalist	3	7.5
No action	3	7.5

^*∗*^Multiple responses were possible.

## Data Availability

The data used to support the findings of this study are included within the article.
